# Empathy in Hippocampal Amnesia

**DOI:** 10.3389/fpsyg.2013.00069

**Published:** 2013-03-22

**Authors:** J. N. Beadle, D. Tranel, N. J. Cohen, M. C. Duff

**Affiliations:** ^1^Department of Psychiatry, University of Iowa College of MedicineIowa City, IA, USA; ^2^Department of Neurology, Division of Cognitive Neuroscience, University of Iowa College of MedicineIowa City, IA, USA; ^3^Beckman Institute, University of Illinois at Urbana-ChampaignIL, USA; ^4^Department of Communication Sciences and Disorders, University of IowaIowa City, IA, USA

**Keywords:** hippocampus, declarative memory, social cognition, empathy

## Abstract

Empathy is critical to the quality of our relationships with others and plays an important role in life satisfaction and well-being. The scientific investigation of empathy has focused on characterizing its cognitive and neural substrates, and has pointed to the importance of a network of brain regions involved in emotional experience and perspective taking (e.g., ventromedial prefrontal cortex, amygdala, anterior insula, cingulate). While the hippocampus has rarely been the focus of empathy research, the hallmark properties of the hippocampal declarative memory system (e.g., representational flexibility, relational binding, on-line processing capacity) make it well-suited to meet some of the crucial demands of empathy, and a careful investigation of this possibility could make a significant contribution to the neuroscientific understanding of empathy. The present study is a preliminary investigation of the role of the hippocampal declarative memory system in empathy. Participants were three patients (1 female) with focal, bilateral hippocampal (HC) damage and severe declarative memory impairments and three healthy demographically matched comparison participants. Empathy was measured as a trait through a battery of gold standard questionnaires and through on-line ratings and prosocial behavior in response to a series of empathy inductions. Patients with hippocampal amnesia reported lower cognitive and emotional trait empathy than healthy comparison participants. Unlike healthy comparison participants, in response to the empathy inductions hippocampal patients reported no increase in empathy ratings or prosocial behavior. The results provide preliminary evidence for a role for hippocampal declarative memory in empathy.

## Introduction

The scientific investigation of empathy has become a cornerstone of the study of social cognition (Preston and de Waal, 2002; Adolphs, 2003). Indeed, empathy, or the ability to understand and share the feelings of others, is integral to the quality of our relationships with others and plays an important role in relationship satisfaction and subjective well-being (Davis and Oathout, 1987; Wei et al., 2011). Converging methods point to a network of structures that contribute to empathy including the bilateral orbitofrontal and ventromedial prefrontal cortices (vmPFC), anterior cingulate, anterior insula, and amygdala (Eslinger, 1998; Mesulam, 2000; Hornak et al., 2003; Shamay-Tsoory et al., 2003, 2009; Singer et al., 2004; Anderson et al., 2006; Lamm et al., 2007; Beadle, 2011).

The purpose of the current study is to investigate the contribution of the hippocampus to empathy. Whereas the hippocampus and its core processing features have not previously been a focus of empathy research, our motivation for their inclusion is based on the role of the hippocampus in declarative (relational) memory (Cohen and Eichenbaum, 1993; Eichenbaum and Cohen, 2001) and a set of empirical findings with neurological patients with hippocampal amnesia pointing to deficits in various aspects of social behavior. In addition, we have heard anecdotal reports from the family members of patients with amnesia that these individuals have post-morbid changes in the ability to understand or predict the thoughts and feelings of others, and to display compassion or offer help to others in need, suggesting there may be disruptions in empathy and prosocial behavior. Our overarching hypothesis, elaborated below, is that the hippocampus is intimately tied to a set of cognitive processes, and is a critical component of the network of brain structures that supports aspects of social cognition. Here we extend this proposal to examine the contribution of the hippocampus to empathy.

### Hippocampal declarative memory and empathy

The role of the hippocampus (and related medial temporal lobe structures) in the formation of new long-term memories and their subsequent retrieval is well established. The hippocampus also plays a critical role in support of declarative memory *use*. The hippocampal declarative memory system has two processing features or properties that are critical to our hypothesis for its role in social cognition. First, declarative memory supports the flexible expression of memory (O’Keefe and Nadel, 1978; Cohen, 1984; Squire, 1992; Cohen and Eichenbaum, 1993; Bunsey and Eichenbaum, 1996; Dusek and Eichenbaum, 1997; Eichenbaum and Cohen, 2001). The *representational flexibility* of this form of memory permits it to be accessible across processing systems (as when a rich, multisensory autobiographical memory is evoked by the sight of a familiar face or the sound of a familiar song) and to be used in novel situations. This flexibility permits memory to be called upon promiscuously in supporting diverse and complex cognitive and social capabilities. The second property of the hippocampal declarative memory system is its support of *relational memory binding* (Cohen and Eichenbaum, 1993; Cohen et al., 1997; Ryan et al., 2000; Eichenbaum and Cohen, 2001; Davachi, 2006), which permits the encoding of memories of (even arbitrary) co-occurrences of people, places, and things along with the spatial, temporal, and interactional relations among them (see Konkel et al., 2008), that constitute events, as well as representations of relationships among events, providing the basis for the larger record of one’s experience.

Recent evidence has also emerged that the hippocampus plays a role in on-line processing. Although most research on hippocampal declarative memory, as with other forms of long-term memory, has been on its role in the formation of new memories and subsequent recollection, recent work by our lab and others (Hannula et al., 2006; Olson et al., 2006; Barense et al., 2007; Hannula and Ranganath, 2008; Warren et al., 2010) show that it is also critical for on-line processing, i.e., for *acting on the present for the present*. The declarative memory system, via hippocampal-cortical connections, is in ongoing interaction with various cortical processing areas, as new information is perceived, as old information is retrieved, and as processing outcomes are held on-line to be evaluated, manipulated, and used in service of behavioral performance. Taken together, the representational flexibility, relational memory binding, and on-line processing afforded by the hippocampus in support of declarative memory use may also support the capability to manage the very complex social relationships and social interactions that are a necessary part of successful social functioning in the world.

Indeed, a growing body of research points to the hippocampus and the declarative memory system as being important for various types of social behavior. For instance, we have shown that hippocampal amnesia impairs decision-making (Gupta et al., 2009a), character judgments (Croft et al., 2010), and various aspects of language and social discourse (Duff and Brown-Schmidt, 2012). Patients with amnesia may also have difficulty establishing and maintaining interpersonal relationships as they have smaller social networks than age and sex matched healthy comparison participants (Gupta et al., 2009b; Davidson et al., 2012). These studies suggest that deficits in the cognitive processes supported by the hippocampus (e.g., representational flexibility, relational binding, on-line processing) disrupt a core set of abilities necessary for recognizing the shifting and changing status of friends and foes, thinking about ourselves and other people, and communicating events in the moment from time frames that stretch from the distant past to possible futures. The extent to which these same hippocampal dependent processes are also important for empathy is an open question.

Empathy is defined by its cognitive and emotional components (Davis, 1980, 1983; Batson, 1991; Eisenberg et al., 1994; Preston and de Waal, 2002). The cognitive component of empathy supports our ability to understand the mental states of another person, including their thoughts, intentions, and feelings. This involves *perspective-taking* which entails imagining or simulating another person’s mental state. Perspective taking is thought to involve the flexible re-experiencing of relevant autobiographical memories or semantic social knowledge about the situation or individual. The emotional component of empathy supports our ability to feel sympathy or compassion for another individual in need and has been termed *empathic concern*. Empathic concern may involve the processes of *emotion contagion* and *emotional responsiveness*, enabling individuals to vicariously experience the emotions of another person. Importantly, individuals often employ emotion regulation in order to dampen their negative emotional arousal due to experiencing others’ vicarious emotions (i.e., *personal distress*) ultimately leading to the experience of *empathic concern*.

While we are agnostic regarding the main theories of empathy, we speculate that the hippocampus and its related processes could play a role in each. There are three main theories of empathy: (1) theory–theory, (2) simulation theory, and an (3) adapted simulation theory. Theory–theory purports that we discern others’ mental states by developing a theory about their behavior (Gopnik and Wellman, 1992, 1994; Gopnik and Meltzoff, 1997). The development of a theory about others’ mental states may involve the hippocampus to bind together social and emotional information about the other person, the scenario, and the environmental location and to hold this information on-line to make judgments and comparisons. Such a role for the hippocampus would, in part, be consistent with neuroimaging evidence suggesting hippocampal recruitment in theory of mind, or the cognitive domain of empathy (Buckner and Carroll, 2007; Spreng et al., 2009; Spreng and Mar, 2012). Whereas theory of mind is often linked to the frontal lobes, Buckner and Carroll (2007) proposed that the processes by which we project ourselves into a different time and place to remember our past are the same processes by which we project ourselves into the future or into the mental states of others and this process may involve the hippocampus. This projection of self into another person’s mental state may reflect the process of *perspective-taking* that occurs in the cognitive component of empathy. Additional evidence that the hippocampus is important for self-projection comes from a study that showed that patients with hippocampal amnesia have difficulty imagining future events (Hassabis et al., 2007). Other studies show that the hippocampus is involved in tasks that require the flexible re-construction of previous memories or imagination of either new events in the future or others’ mental states (Spreng et al., 2009; Spreng and Grady, 2010; Spreng and Mar, 2012).

Simulation theory suggests that the way in which we are able to understand another person’s mental state is through internal simulation that occurs after we first comprehend their mental state (Gordon, 1986; Heal, 1986). This pre-requisite understanding of others’ mental states may recruit the hippocampus in a manner similar to that for theory-theory. However, in order for an individual to feel *empathic concern* for others who are suffering rather than *personal distress*, individuals must regulate their emotions and distinguish others’ emotions from one’s own. This process may recruit the hippocampus as individuals must maintain on-line their shared emotional experience, distinguish the relational emotion-individual pairings experienced by the self and other, and flexibly use personal memories to guide behavior in the moment. It is also possible that certain social demands disproportionately engage the hippocampus. Rabin and Rosenbaum (2012) demonstrated that imagining or simulating the mental state of familiar or known individuals recruits the hippocampus to a greater degree than for unknown individuals. The adaptation of simulation theory defines the internal simulation process to be unconscious and automatic rather than a conscious process requiring knowledge about the mental state of the other person (Gallese, 2003; Goldman and Sripada, 2005; Gazzaniga, 2008). Considering this theory in the context of emotional empathy and its subdomains would suggest that the process by which individuals vicariously adopt the emotions of others (i.e., *emotion contagion* and *emotional responsiveness*) would occur automatically and would not require intact theory of mind. The hippocampus may be recruited to maintain on-line and flexibly use emotional information about one’s own emotional state and that of the other person to experience empathy toward them. Previous research has demonstrated that the hippocampus is involved in unconscious processing of relational information (Ryan et al., 2000; Hannula and Ranganath, 2009; Hannula and Green, 2012).

### The current study

The current study extends our proposal about the role of the hippocampus in social cognition to empathy. We conducted a preliminary investigation of cognitive and emotional empathy in patients with hippocampal damage and severe declarative memory impairment. This consisted of an assessment of the subdomains of empathy including *perspective-taking*, *emotion contagion*, *emotional responsiveness*, and *empathic concern*. As a first pass at measuring empathy in amnesia, we used the gold standard tools from the field of social neuroscience, i.e., self-report questionnaires. Building on our success using an emotion induction paradigm in this same group of hippocampal patients (Feinstein et al., 2010), we investigated the induction of empathy through two methods adapted from standard empathy methodology (Batson et al., 1995; Batson and Moran, 1999). We hypothesized that patients with hippocampal amnesia would report lower empathy on trait questionnaires and measures of empathy after undergoing an empathy induction than healthy comparison subjects.

## Materials and Methods

### Experiment 1

#### Participants

Participants were three patients (one female) with bilateral damage to the hippocampus and severe declarative memory impairment and three healthy comparison participants matched pair-wise to the amnesic patients on age, sex, handedness, and education (see Table 1). The etiology for the patients was an anoxic/hypoxic event (e.g., cardiac arrest, status epilepticus).

**Table 1:** **Demographic and neuropsychological characteristics of hippocampal patients**.

Patient	Sex	Onset age (years)	Testing age (years)	Edu (years)	Chronicity (years)	WAIS-III FSIQ	WMS-III GMI	Token test	Boston naming test	BDI
1846	F	30	46	14	16	84	57	41	43	9
2563	M	45	54	16	9	94	63	44	52	0
2363	M	42	53	18	11	98	73	44	58	0
Mean (SD)		39.0 (7.9)	51.0 (4.4)	16.0 (2.0)	12.0 (3.6)	92.0 (7.2)	64.3 (7.2)	43.0 (1.7)	51.0 (7.5)	3 (5.2)

*M, male; F, female; Edu, education; WAIS-III, Wechsler Adult Intelligence Scale-III; FSIQ, Full Scale Intelligence Quotient; WMS-III, Wechsler Memory Scale-III; GMI, General Memory Index; BDI, Beck Depression Inventory*.

For patients 2363 and 1846, structural magnetic resonance image (MRI) examination was completed confirming bilateral hippocampal damage and significantly reduced hippocampal volumes [studentized residual differences in hippocampal volume relative to a matched comparison group were −2.6 and −4.24 *z*-scores, respectively; (Allen et al., 2006)]. There was no evidence of damage to the structure of the amygdala in either 1846 or 2363 (Allen et al., 2006; Warren et al., 2012). We do not have functional connectivity data on these patients so we cannot comment on the integrity of the functional connectivity between the hippocampus and related structures. For patient 2563, anatomical analysis was based on computerized tomography (he wears a pacemaker and was unable to undergo MRI) and only damage in the hippocampal region was visible.

Neuropsychological testing confirmed a selective and severe memory impairment disproportionate to any deficits in general cognitive or intellectual functioning (see Table 1). Performance on the Wechsler Memory Scale-III (General Memory Index) was at least 25 points lower than performance on the Wechsler Adult Intelligence Scale-III (Full Scale IQ; *M*=92.0), with an average delay score on the memory scale (64.3) that was nearly 3 standard deviations below population means. Basic speech and language abilities were intact and all patients performed within normal limits on standardized measures of language. Patients 2363 and 2563 indicated no evidence of depression on the Beck Depression Inventory (BDI). Responses on the BDI from 1846, the only female participant, were interpreted as mild depression (see Warren et al., 2012 for more information about 1846’s case).

#### Measures

Empathy is multidimensional in nature and includes cognitive and emotional components. Empathy was assessed using four self-report, trait measures including the Interpersonal Reactivity Index (IRI; Davis, 1980), the Questionnaire Measure of Empathic Tendency (QMET; Mehrabian and Epstein, 1972), the Empathy Quotient (EQ; Baron-Cohen and Wheelwright, 2004), and the Emotion Contagion Scale (EC; Doherty, 1997). While the IRI and EQ assess both components of empathy, the QMET and EC focus on the emotional component of empathy. These questionnaires have demonstrated high validity and reliability in healthy adult samples (for questionnaire details see below). Two of the patients (1846 and 2363) were available to complete the questionnaires for a second time at least 6 months after the initial testing session allowing an assessment of test reliability. In general, this revealed adequate reliability across testing sessions (see Table 2).

**Table 2:** **Trait empathy: reliability**.

Measures	1846	1846 Re-test	Diff score	2363	2363 Re-test	Diff score
IRI-PT	16	15	1	17	21	−4
IRI-EC	19	23	−4	19	19	0
IRI-FS	7	11	−4	21	19	2
IRI-PD	19	17	2	12	12	0
EQ-sum	47	46	1	42	50	−8
EQ-cog	11	10	1	11	11	0
EQ-emot	14	13	1	7	11	−4
EQ-soc	6	5	1	5	7	−2
QMET	36	54	−18	17	19	−2
EC	2.93	3.33	−.40	2.53	2.73	−.20

*IRI-PT, Interpersonal Reactivity Index (IRI)-Perspective-Taking Subscale; IRI-EC, IRI Empathic Concern subscale; IRI-FS, IRI Fantasy subscale; IRI-PD, IRI Personal Distress Subscale; EQ, Empathy Quotient (EQ); EQ-Sum, total score on EQ; EQ-Cog, EQ Cognitive Empathy Factor; EQ-Emot, EQ Emotional Reactivity Factor; EQ-Soc, EQ Social Skills Factor; QMET, Questionnaire Measure of Empathic Tendency; EC, Emotion Contagion Scale; Diff score, Initial testing – Re-test score*.

Participant data on the trait questionnaires was compared to the healthy comparison participants from the present study (see Table 3). As a secondary comparison, the participant data was also compared with healthy control normative data available in the literature. A standardized *z* score was computed for each participant that compared their raw score on the questionnaire to the mean and standard deviation of the normative data set. Normative data sets for these questionnaires consisted of individuals in the young adulthood age range and participants were compared based upon gender-specific norms. A *z* score was computed for the patient with hippocampal damage as well as the family member and healthy comparison participant. This is particularly important in the case of our dataset because our present sample is somewhat older than the young adulthood range found in most normative studies. The comparison data sets included: (Davis, 1980; IRI), (Lawrence et al., 2004; EQ sum score), (Lawrence et al., 2007; EQ factor scores), (Mehrabian and Epstein, 1972; QMET), and (Doherty, 1997; EC). When available, a qualitative comparison of the participant data was made with an older adult sample that was similar in age to the participants.

**Table 3:** **Trait empathy**.

Participant			Measure									
			IRI				EQ				QMET	EC
			PT	EC	FS	PD	Total	Cog	Emot	Soc	Total	Total
1846	Patient	Raw	16	19	7	19	47	11	14	6	36	2.93
		*z*	−.40	−.70	−2.27	1.34	−.39	2.33	3.95	−.16	−.38	−1.46
	Family	Raw	7	16	2	19	25	2	5	4	16	2.47
		*z*	−2.26	−1.48	−3.24	1.34	−2.78	−2.67	−.79	−.96	−1.33	−2.31
	NC	Raw	24	22	14	16	51	13	10	9	83	3.00
		*Z*	1.25	.09	−.92	.74	.04	3.44	1.84	1.04	1.86	−1.33
	P–F score		9	3	5	0	22	9	9	2	20	.46
2363	Patient	Raw	17	19	21	12	42	11	7	5	17	2.53
		*z*	.05	−.01	.94	.56	.07	3.39	.72	−.40	−.27	−1.60
	Family	Raw	16	25	13	16	29	4	9	3	20	2.20
		*z*	−.17	1.42	−.49	1.44	−1.22	−.50	1.41	−1.20	−.14	−2.23
	NC	Raw	21	24	19	11	55	10	14	5	86	3.53
		*z*	.89	1.18	.58	.34	1.36	2.83	3.14	−.40	2.86	.33
	P–F score		1	−6	8	−4	13	7	−2	2	−3	.33
2563	Patient	Raw	11	23	7	1	51	9	12	10	14	2.13
		*z*	−1.22	.94	−1.56	−1.86	.96	2.28	2.45	1.60	−.41	−2.37
	NC	Raw	21	23	25	22	59	12	16	8	53	2.93
		*z*	.89	.94	1.66	2.76	1.75	3.94	3.83	.80	1.36	−.83
HC		M	14.67	20.33	11.67	10.67	46.67	10.33	11.00	7.00	22.33	2.53
GRP		SD	3.21	2.31	8.08	9.07	4.51	1.15	3.61	2.65	11.93	.40
NC		M	22.00	23.00	19.33	16.33	55.00	11.67	13.33	7.33	74.00	3.15
GRP		SD	1.73	1.00	5.51	5.51	4.00	1.53	3.06	2.08	18.25	.33

*HC GRP, group of patients with hippocampal damage; NC GRP, group of normal healthy comparison participants; NC, each normal, healthy comparison participant matched to a particular patient; Raw, raw sum score on each questionnaire; Z, standardized score on each questionnaire based upon the mean and standard deviation of a normative control sample from the literature; P–F score, Patient–Family member raw score on questionnaire; IRI, Interpersonal Reactivity Index (IRI); PT, Perspective-Taking Subscale of IRI; EC, Empathic Concern subscale of IRI; FS, Fantasy subscale of IRI; PD, Personal Distress Subscale of IRI; EQ, Empathy Quotient; Sum, total score on EQ; Cog, Cognitive Empathy Factor of EQ; Emot, Emotional Reactivity Factor of EQ; Soc, Social Skills Factor of EQ; QMET, Questionnaire Measure of Empathic Tendency; EC, Emotion Contagion Scale*.

##### Interpersonal Reactivity Index

The IRI (Davis, 1980) is one of the few empathy questionnaires designed to measure empathy as a multidimensional construct in healthy adults. The IRI has four subscales measuring an individual’s perception of their ability in each of these domains: Perspective Taking (PT-adopting the mental perspective of another person), Empathic Concern (EC-experiencing feelings of compassion for others), Fantasy (FS-adopting the perspective of a fictional character in a book or movie), and Personal Distress (PD-feeling unease or distress in the face of the physical or emotional harm of another person). Each subscale contains 7 items that are summed to create a total score for each subscale with ranges from 0 to 28 points, with higher scores indicating greater empathy. The IRI has adequate test/re-test reliability across its four subscales in healthy adults (range: *r*=.61 to .81; test/re-test interval: 60–75 days) and the subscales have adequate internal consistency (PT: Cronbach’s α: males =.71, females =.75; EC: Cronbach’s α: males =.68, females =.73; FS: Cronbach’s α: males =.78, females =.79; PD: Cronbach’s α: males =.77, females =.75.)

Cognitive empathy is measured by the PT subscale through such items as, “When I’m upset at someone, I usually try to ‘put myself in his shoes’ for awhile.” An example item from the FS subscale includes, “When I am reading an interesting story or novel, I imagine how I would feel if the events in the story were happening to me.” For the EC subscale an example item includes, “I often have tender, concerned feelings for people less fortunate than me.” The PD subscale measures vicarious negative arousal resulting from viewing another person’s emotional or physical distress, for example, “I sometimes feel helpless when I am in the middle of a very emotional situation.”

##### Empathy Quotient

The Empathy Quotient (EQ) was developed to assess dispositional empathy in individuals with Asperger’s Syndrome/High Functioning Autism and in healthy adults (Baron-Cohen and Wheelwright, 2004). The scale has sufficient test/re-test reliability in healthy adults (*r*=.84; test/re-test interval: 10–12 months; Lawrence et al., 2004) and adequate internal validity (Cronbach’s α=.92; Baron-Cohen and Wheelwright, 2004). Although originally designed to measure empathy as a uni-dimensional construct, a recent factor analysis has revealed that it may measure three factors: Cognitive Empathy, Emotional Reactivity, and Social Skills (Lawrence et al., 2004). Total scores are computed by summing responses to all of the items, while factor scores involve summing items that measure a particular factor. The range of total scores is from 0 to 80 points, with higher scores indicating greater empathy. While cognitive empathy is assessed by the Cognitive Empathy Factor and includes such items as, “I am good at predicting how someone will feel,” emotional empathy is measured by the Emotional Reactivity factor through items such as, “I get upset if I see people suffering on news programs.” Emotional empathy as measured by this scale is more similar to the construct of emotion contagion than sympathy. The Social Skills factor does not measure empathy but rather assesses individuals’ perceptions of their ability to interact socially with others, for example, “I find it hard to know what to do in a social situation” (which is a reverse-scored item).

##### Questionnaire Measure of Empathic Tendency

The Questionnaire Measure of Empathic Tendency (QMET) was designed to measure dispositional emotional empathy in healthy adults (Mehrabian and Epstein, 1972). The split-half reliability for this scale in healthy adults is .84. The QMET has demonstrated good construct validity in healthy adults through its association with other measures of empathy such as the IRI-EC [males: *r*(225)=.63, *p*<.05, females: *r*(235)=.56, *p*<.05] and the IRI-PT [males: *r*(225)=.22, *p*<.05; females: *r*(235)=.17, *p*<.05; Davis, 1983]. Because the scale measures vicarious emotional responsiveness to others, it is more similar to the emotion contagion construct than to sympathy. Emotional empathy is assessed by computing a total sum score on the thirty-three items on the questionnaire, some of which are negatively worded and thus reverse scored. The response scale ranges from −4 (“I strongly disagree”) to +4 (“I strongly agree”) and is anchored at 0 with a range of scores from −132 (lowest empathy) to +132 points (highest empathy). Although initially designed to be separated into subscales, it was shown that the best fit is a uni-dimensional measure of emotional empathy. A couple of example items include: “I tend to get emotionally involved with a friend’s problems,” and, “Seeing people cry upsets me.”

##### Emotion Contagion Scale

The Emotion Contagion Scale (EC) measures one’s tendency to vicariously experience the emotions of others in daily life and measures emotion contagion (Doherty, 1997). In healthy adults, this scale has shown adequate test-retest reliability [*r*(41)=.84, *p*<.001, over a three week interval] and high internal consistency (Cronbach’s α=.90). The EC scale shows moderate associations with other measures of emotional empathy, such as the IRI-EC [*r*(119)=.37, *p*<.05] and the QMET [*r*(80)=.47, *p*<.05]. The EC scale measures emotion contagion as a uni-dimensional construct through fifteen positively scored items. The response choices range from 1 (“Never”), 2 (“Rarely”), 3 (“Often”), and 4 (“Always”), and the average score of the items is computed, with higher scores indicating greater emotion contagion. A few example items from the scale are, “If someone I’m talking with begins to cry, I get teary-eyed” and, “I get filled with sorrow when people talk about the death of their loved ones.”

## Results

### Trait cognitive empathy

On the Perspective Taking subscale of the IRI (IRI-PT), which assesses one’s ability to adopt the mental perspective of others, all three hippocampal participants (1846, 2363, 2563) reported lower scores on this measure than healthy comparison participants (see Table 3). In fact, the patients’ ratings were 3 standard deviations (SD) or more below that of the three demographically matched healthy comparison participants (1846: 3.47 SD; 2363: 2.89 SD; 2563: 6.35 SD). The group of hippocampal patients were significantly different from the healthy comparison group on this measure [Mann–Whitney: *z*(4)=1.99, *p*<.05]. The family members of two of the amnesic patients (1846 and 2363) reported the patients as having even lower PT scores than the patients themselves reported (difference score: patient score – family member score: 1846 = 9 points; 2363 = 1 point). We were unable to obtain family member ratings for patient 2563.

Next, we compared the participants’ scores on the IRI-PT to a normative dataset of healthy adults (Davis, 1980). All of the matched comparison participants had positive *z* scores ranging from approximately +1 SD to +1.25. In comparison, the patients’ *z* scores were negative in 2 out of 3 cases (1846: −.40, 2563: −1.22, 2363: .05). Furthermore, where family member scores were available, their *z* scores were more negative than the reports of the patients (1846: −2.26, 2363: −.17). In an effort to also compare the patients to a more similar age cohort, the patients’ responses were compared to a previous paper examining trait empathy in older adults (age: *M*=66.2, *SD*= 7.6; range = 55–81 years; Beadle et al., 2012). The patients with hippocampal damage in the present study were slightly younger (*M*=51, *SD*=4.4 years). This previous paper demonstrated that older adults report lower cognitive empathy than younger adults (Beadle et al., 2012). For this comparison, the hippocampal group was approximately 1 SD below the group of older adults, suggesting that their reported scores are not simply an effect of age (hippocampal patients: *M*=14.7; older control group: *N*=20, *M*=17.7, *SD*=3.4).

The EQ cognitive empathy scale measures one’s ability to accurately predict others’ mental states. Two out of three patients with hippocampal amnesia reported lower scores (1846, 2563) than the demographically matched healthy comparison participants on this scale. Relative to the mean of the healthy comparison participants, 2563 was 1.7 SD lower, while 1846’s score was within 1 SD from the comparison group mean. As a group, however, the amnesic patients did not differ from healthy participants on the EQ cognitive empathy factor [*z*(4)=1.11, *p*=.27]. Because the family members of patients 1846 and 2363 reported the patients as having much lower cognitive empathy than the patients’ own ratings (difference score: 1846=9 points; 2363=7 points), it suggests that the patients’ own ratings may not be completely accurate in this case. In comparison to standard normative data (Lawrence et al., 2007), healthy comparison participants had positive *z* scores ranging from +2.83 to +3.94 SD, while the family member ratings of the patients were both negative (1846F:−2.67, 2363F:−.50).

### Trait emotional empathy

Emotional responsiveness to others, one domain of emotional empathy, was reported to be low in all three amnesic participants relative to the healthy comparison participants on the Questionnaire Measure of Empathic Tendency (QMET; see Table 3). The amnesic patients were at least 2 SD lower than the healthy comparison participants’ mean (1846: 2.10 SD, 2363: 3.10 SD, 2563: 3.30 SD), a difference that was marginally significant [*z*(4)=1.96, *p*=.05]. Family members rated the patients as slightly higher than the patients’ own ratings in one case (2363: patient=17, family member=20), and quite a bit lower in another case (1846: patient=36, family member=16). In comparison to a normative data set (Mehrabian and Epstein, 1972), the patients and the family member reports resulted in negative *z* scores (range: patient=−.41 to −.27; family member=−1.33 to −.14). In contrast, healthy comparison participants all had positive *z* scores (range: 1.36 to 2.86).

For the EQ Emotional Reactivity Factor, which also measures emotional responsiveness to others, two out of three amnesic participants were lower than the healthy comparison participants. Of the two hippocampal amnesic participants, one had a score 2 SD away from the mean of the demographically matched healthy comparison participant group (2363) and the other patient was .4 SD lower (2563). As a group, the hippocampal patients did not significantly differ from the demographically matched healthy comparison participants on the EQ Emotional Reactivity Factor [*z*(4)=.89, *p*=.38]. The family member of 2363 rated the patient 2 points higher than the patient’s own rating, while the family member for 1846 rated the patient 9 points lower. Relative to a normative dataset (Lawrence et al., 2007), 2363 and 2563 had qualitatively lower *z* scores than their matched comparison participants (2363=.72, 2363C=3.14; 2563=2.45, 2563C=3.83).

Two out of three participants with amnesia were also low relative to the healthy comparison participants on another measure of emotional responsiveness that assesses primitive emotional contagion toward others (Emotion Contagion Scale), with scores approximately 2 SD or more lower than the healthy comparison participant mean (2363: 1.90 SD; 2563: 3.10 SD). Hippocampal patients as a group did not differ significantly from the demographically matched healthy comparison participants on the EC [*z*(4)=1.77, *p*=.08]. For both 1846 and 2363, the family members of these patients rated them as having lower levels of emotion contagion than the patients themselves reported (1846: patient=2.93, family member=2.47; 2363: patient=2.53, family member=2.20). In terms of the patients’ scores relative to normative data (Doherty, 1997), patients had lower *z* scores than the matched comparison participants (1846=−1.46, 1846C=−1.33, 2363=−1.6, 2363C=.33, 2563=−2.37, 2563C=−.83). Furthermore, patients’ family members *z* scores were even lower than the patients’ (1846F=−2.31, 2363F=−2.23).

Sympathy or compassion toward others in need was measured by the IRI Empathic Concern subscale. On this subscale, two out of three participants with amnesia reported lower scores (1846, 2363) than the healthy comparison participants. The scores of 1846 and 2363 were 4 SD lower than the healthy comparison participants mean. When comparing the group of hippocampal patients to the healthy comparison participants, there were no significant differences [*z*(4)=1.35, *p*=.18]. In terms of the family member ratings from the present study, 1846’s family member reported slightly lower scores than the patient (1846=19, family member=16), while 2363’s family member reported slightly higher scores than the patient (2363=19, family member=25). A comparison to a normative dataset of younger adults (Davis, 1980) revealed that 1846, based upon self and family member ratings, had *z* scores that were lower than that of their matched comparison participant (1846=−.70, 1846F=−1.48, 1846C=.09). The *z* scores for 2363 and 2563 did not show a consistent pattern (2363=−.01, 2363F=1.42, 2363C=1.18; 2563=.94, 2563C=.94). Compared to data from a group of older adults, the mean of the hippocampal group was similar to that of the older adult control mean (hippocampal amnesic: *M*=20.30, healthy older adult control mean: *M*=20.78, *SD*=2.64; Beadle et al., 2012).

### Interim discussion

These results provide preliminary evidence for lower trait cognitive and emotional empathy on some measures in patients with hippocampal amnesia than in healthy comparison participants, both matched to the patients and group norms in the literature. However, given that all of these measures rely on self-report, we were concerned that the extent of the patients’ memory impairment may have influenced their ability to accurately report their experience of cognitive and emotional empathy. These questionnaires likely require some degree of declarative memory to recall how one responded in a particular situation in the past or to imagine how one might respond in the future. The consistency of the patients’ reports alleviate some of these concerns, as do the family member reports of the patients’ empathy. The reports from the patients’ family members were largely consistent with the patients’ own reports of lower empathy. In fact, in line with their anecdotal reports, the family member reports were actually slightly lower than those of the patients in several cases, suggesting an observable deficit in empathy. Furthermore, in addition to age-matched comparison data, we also compared the amnesic patients’ ratings to norms from healthy older adults who are assumed to have experienced age-related changes to hippocampal function. The amnesic patients had even lower levels of empathy than these older adults in the case of cognitive empathy. That said, we were still concerned about the limitations of the questionnaires as well as our low number of participants and the variability across measures (i.e., not all patients were lower on all questionnaires) and sought a more objective measure of empathy.

In a second experiment, we conducted a behavioral study that assessed the induction of empathy and measurement of prosocial behavior in hippocampal amnesia. In previous work in our lab, these same amnesic patients underwent an emotion (happiness, sadness) induction procedure (using affectively-laden film clips) to ascertain whether their experience of a basic emotion would persist beyond their memory for the sadness-inducing films (Feinstein et al., 2010). The induction procedures were successful as the patients reported higher levels of the target emotion long after they were able to recall any information about the film clips. Building upon this previous study, we measured the level of empathy participants reported after undergoing an empathic induction and the prosocial behavior they demonstrated after experiencing the induction.

## Materials and Methods

### Experiment 2

#### Participants

Two participants with hippocampal amnesia from Experiment 1 (1846 and 2363) were available to complete Experiment 2A and B over two separate testing sessions. In Experiment 2A, the behavior of these patients was compared to healthy comparison participants matched to the patients on age, sex, handedness, and education^1^. For Experiment 2B, the performance of the hippocampal amnesic participants was compared to a separate group of 7 healthy comparison participants (4 females, 3 males) matched to the hippocampal patients on age and education.

### Experiment 2A

#### Measures

##### Empathy induction through audio recording

We adapted an empathy induction procedure from Beadle (2011) where participants listened to two audio recordings that included a neutral condition and an empathy condition. In the original version of the procedure, participants were told they were overhearing a conversation between their opponents in an economic game and a research assistant over a speakerphone. Here, participants listened to the exact same recording but were told that they were in fact listening to an audio recording. In pilot studies of these two versions of the task (overhearing vs. recording), we found the two versions to be comparable in their effectiveness in inducing empathy in 12 healthy adults (25–62 years; see Beadle, 2011 for details).

The premise of the audio recordings were that they were taped conversations between a female Research Assistant in her 20’s interacting with a series of two participants in a previously conducted experiment and each of the participants were men of approximately 55 years of age. The Research Assistant made “small-talk” with participants and asked about their day as they were preparing to begin the experiment. In reality, these audio recordings were performed in a sound studio by a series of community theater actors and a graduate student serving in the role of the Research Assistant. In the neutral conversation, a man talks about the events that occurred in his day thus far which included reading the newspaper over breakfast and talking with his wife. The empathy-inducing conversation begins with a man talking about his day and describing how he played a card game, when he reveals that it is the anniversary of his son’s death and demonstrates his deep anguish and grief over his loss.

##### Empathy ratings

Immediately before and after listening to each audio recording, participants completed a self-report questionnaire consisting of items that assessed participants’ current level of empathy, as well as other positive and negative emotions (Beadle, 2011). Adapted from the instructions for the Positive and Negative Affective Schedule (PANAS; Watson and Clark, 1994), participants were asked to perform ratings based upon a series of items following the prompt, “Indicate to what extent you feel this way right now, that is, at the present moment” on a rating scale that ranged from 1 (“very slightly or not at all”) to 5 (“extremely.”) The empathy items on this questionnaire were derived from the emotional response scale that has been used to measure empathic concern in a variety of empathy induction studies in young adults (Batson, 1987, 1991). The specific items that measured empathy in the present study included “sympathetic” and “compassionate.” Also drawn from Batson and colleagues emotional response scale were items measuring the social emotion of personal distress. In addition, items were drawn from the PANAS that assessed sadness, hostility, and joviality (Watson and Clark, 1994). Two items were used to measure each domain of emotion (empathy, sadness, joviality, hostility, and personal distress), and then within each individual the two responses were averaged. To determine the level of empathy specifically produced by the empathy induction, a change score was calculated that accounted for baseline ratings prior to each induction as well as due to the neutral condition [Empathy Change Score = (After – Before Empathy Induction) – (After – Before Neutral Induction)].

### Experiment 2B

#### Measures

##### Empathy induction through note

The second experiment (Part B) also involved an empathy induction, but in this case empathy was induced in participants through a written note rather than an audio recording. In addition, participants’ prosocial behavior was measured in response to the empathy induction. Participants were told that the purpose of the study was to play an economic game against two other participants, one at a time. This paradigm was employed in a previous study of healthy younger and older adults (Beadle et al., under review). Out of the three participants, two would serve in the role of the “Sender,” meaning that they would be asked to write a note about something interesting that recently happened to them, and one of them would be in the role of the “Receiver” who would read the notes from the opponents immediately prior to playing them in a series of economic games. The use of notes to induce empathy in an economic game context was adapted from methodology by Batson and colleagues (Batson et al., 1995; Batson and Moran, 1999), while the specific content of the notes used in this experiment was the same as that used by Beadle and colleagues (Beadle et al., under review). The content of the empathy induction note consisted of the participant describing that they recently found out they have a potentially fatal form of skin cancer, and their thoughts and feelings as they attempted to cope with this news. In the neutral note, the participant describes a series of errands they completed in the downtown area, and then in order to make the arousal level more similar to that of the empathy note, the participant discusses being followed on the freeway by another car that eventually drives away. The participants performed empathy ratings immediately prior to reading each note and immediately afterward. The self-report scale used in this experiment was the same as the one described in Experiment 2A.

##### Empathy and prosocial behavior

Prosocial behavior was measured as the amount of money ($) given to a game opponent on a standard economic game, the Dictator Game, in response to the neutral condition and the empathy condition. In the Dictator Game, the participant must decide how they would like to split $10 with their game opponent, and their opponent must accept any offer amount. The participant who is splitting the money must follow a few rules: (1) the split must add up to $10; (2) the offer must be in dollar increments; and (3) the offer must range between $1–9. Behavior on the dictator game due to the empathy condition was compared to the control neutral condition.

## Results

### Empathy ratings

#### Empathy induction through audio recording

In comparing the empathy ratings in response to the neutral and empathy conditions, 2363 and 1846 had an empathy change score of 0, indicating that the empathy condition did not produce greater empathy ratings than the neutral condition in these patients (see Table 4). The healthy comparison participant matched to 1846 showed an empathy change score of a 2 point increase due to the empathy induction (vs. the neutral induction) and the healthy comparison participant for 2363 showed an increase in empathy of 1 point.

**Table 4:** **Empathy inductions: ratings**.

Rating	Exp. type	Neutral condition					Empathy condition				
		1846	1846C	2363	2363C	NC M (SD)	1846	1846C	2363	2363C	NC M (SD)
EC	Recording	.50	.00	.00	.00	.00 (.00)	.50	2.00	.00	1.00	1.50 (.71)
	Note	−.50	NA	.50	NA	−.36 (1.21)	−.50	NA	−1.00	NA	1.07 (.67)
HOS	Recording	.00	.00	.00	.00	.00 (.00)	.50	.00	.00	.00	.00 (.00)
	Note	.00	NA	.00	NA	.50 (1.12)	.00	NA	.00	NA	.14 (.38)
JOV	Recording	−1.00	.00	.00	.00	.00 (.00)	−1.50	−.50	−3.00	−.50	−.50 (.00)
	Note	.00	NA	.00	NA	−.64 (1.14)	−.50	NA	.00	NA	−1.00 (.96)
PD	Recording	−1.00	.00	.00	.00	.00 (.00)	−.50	.00	1.00	.00	.00 (.00)
	Note	.00	NA	.00	NA	.50 (1.00)	−1.00	NA	.00	NA	1.21 (1.58)
SAD	Recording	−.50	.00	.00	.00	.00 (.00)	1.00	1.00	1.00	.50	.75 (.35)
	Note	.00	NA	.00	NA	−.07 (.73)	−1.00	NA	.00	NA	1.29 (1.11)

*Values represent change scores of participants’ self-report ratings completed immediately before and immediately after each type of induction. Change score, Rating After – Before Condition (Neutral or Empathy); EC, empathic concern subscale; HOS, hostility subscale; JOV, joviality subscale; PD, personal distress subscale; SAD, sadness subscale; Exp., Experiment; C, healthy matched comparison participant rating; NC, for recording study: represents the mean of the normal, healthy matched comparison participants (*N*=2). For note study: represents the mean of the healthy comparison group (*N*=7). NA, not applicable*.

#### Empathy induction through note

Participants with hippocampal amnesia reported experiencing lower empathy after undergoing an empathy induction (see Table 4) than a group of healthy comparison participants. Specifically, 1846 had a change score of 0 from her ratings on the empathy condition vs. the neutral condition, and this was less than 1 SD away from the healthy comparison group mean (*M*=1.43, *SD*=1.59, Median=1). Participant 2363’s change score included a decrease of 1.5 points in empathy in comparison to the neutral condition and this was approximately 2 SD’s away from the healthy comparison group mean.

### Empathy and prosocial behavior

Hippocampal amnesic participants gave no more money to their game opponent in the neutral condition than in the empathy condition, and this differed from the group of healthy comparison participants who gave more money in the empathy condition than the neutral condition. 1846 gave $4 in the neutral condition and $4 in the empathy condition, while 2363 offered $6 in the neutral condition and $6 in the empathy condition. The healthy comparison participants gave on average $4.14 in the neutral condition (*SD*=1.68) and $5.71 in the empathy condition (*SD*=1.25). 1846 and 2363 showed no difference between the amount of money they gave in the neutral and empathy conditions ($0 change score), while the healthy comparison group gave on average $1.57 (*SD*=1.62, Median=$2) more in the empathy condition than in the neutral condition. The hippocampal amnesic participants’ responses were about 1 SD away from the healthy comparison group mean.

## Discussion

We propose that the hippocampus is intimately tied to a set of cognitive processes, and is a critical component of the network of brain structures, that supports aspects of social cognition. Here, we extended this proposal to test a hypothesis about the role of the hippocampus in empathy. We found preliminary evidence that hippocampal amnesia is associated with reduced cognitive and emotional empathy relative to healthy comparison participants. Patients with hippocampal amnesia had lower ratings of trait empathy on self-report questionnaires of both cognitive and emotional empathy when compared to healthy demographically matched participants, normative data of healthy younger adults, and to older adults who are assumed to have experienced age-related changes to hippocampal function. These self-ratings were consistent with family member ratings, which were either equal to or lower than that of the patients themselves in the majority of cases. Results from the behavioral empathy studies corroborate the self-report data providing preliminary evidence that the hippocampus may be necessary for some aspects of healthy empathy.

A growing body of work suggests that hippocampal memory processes contribute to a variety of other cognitive domains. At the heart of proposals regarding how the hippocampus contributes to capabilities as diverse as visual perception, problem solving, and language processing, to name a few, is the functionality and core processing features of the hippocampus. Here we have expanded the reach of the hippocampus to the neural network that supports social cognition and empathy. Linking disruptions in empathy to the hippocampus demonstrates how promiscuously the hallmark processing features of the hippocampus are used in service of a variety of cognitive domains.

Like other complex behaviors, empathy requires the coordination and orchestration of cognitive and neural systems. Empathy is characterized by the fact that its diverse components must function together in a relational, flexible, and on-line manner in order for effective empathic responding to occur. For instance, in terms of cognitive empathy, the process of perspective-taking necessitates the flexible integration and retrieval of previously acquired memories and social knowledge relevant to the situation in order to imagine and predict the mental state of another person. For emotional empathy, an important interplay must occur between an individual’s vicarious emotional response to another person’s emotional state and adequate emotion regulation of that state in order to experience empathic concern for the other person. This process is likely to require on-line monitoring and updating of one’s own and others’ emotional states. We have argued that the functionality of the hippocampus, in its support of relational binding, representational flexibility, and on-line processing, is well suited to meeting the demands of some aspects of empathy.

While the inclusion of the hippocampus to the neural network of social cognition is supported by our preliminary results on empathy disruptions in patients with hippocampal amnesia, it also raises a number of interesting and open questions about the nature and timing of hippocampal contributions and the interactions with the rest of the network. It will be informative in future work to differentiate the contributions of distinct neural and cognitive systems to empathy and the patterns of impairments in patients with selective damage across the empathy network. For example, with respect to hippocampal contributions, the ability to recognize, process, and experience basic emotions appears to be independent of the hippocampus and instead would be the purview of other neural systems (e.g., amygdala). However, once the processing demands of empathy require the binding of this information to a person and a context, the continuous updating of information, and the flexible deployment of this information in dynamic and evolving situations, patients with hippocampal amnesia are particularly challenged.

While it makes good sense that hippocampal damage and profound amnesia would significantly impair the acquisition and updating of new social information, can the patients rely on their remote episodic and semantic knowledge, that is often judged to be intact, to produce an appropriate empathic response in some contexts? The patients would certainly be in a better position to marshal information from their remote autobiographical memory in service of producing an appropriate empathic response than if they had to rely exclusively on information acquired in the recent past, in the window of their anterograde amnesia. The question, however, is would that remote knowledge be sufficient? And, would the result be the same as someone without hippocampal damage? While we are unaware of any evidence that directly addresses this, we would speculate that the patients with hippocampal amnesia would still be disadvantaged and would not produce a fully normal empathic response relying on remote autobiographical memory alone. One reason for this line of thinking is that the hippocampus plays a role in the flexible manipulation and use of declarative memory. That is, even if representations of a previous empathic response were retrieved from memory, there are still hippocampal contributions, as the use of the reconstructed episode would require the active maintenance of relational information as judgments and comparisons are made between the previous and current situation. These are certainly interesting and open questions for future work on empathy and on the nature of preserved episodic memory more broadly.

Recent literature has suggested that during the process of perspective-taking the hippocampus may show greater recruitment when thinking about the mental states of others who are more similar to us (or known) or in situations that we have encountered (Perry et al., 2011; Rabin and Rosenbaum, 2012). It is thought that the hippocampus may show greater recruitment because the individual may have more relevant personal memories that can serve as a guide in predicting the mental state of a known other (Rabin and Rosenbaum, 2012). On the other hand, there is some evidence for a reduced role for the hippocampus in understanding the mental states of strangers (Rosenbaum et al., 2007). While this question was not addressed specifically in our study, there is some evidence for disruption in thinking about the mental states of close others (as measured by the questionnaires) and strangers (as measured by the empathy induction). Anecdotal reports by the family members about their ability to detect others’ mental states, suggest that they have difficulty detecting the thoughts and feelings of their family members. Given the nature of the measures and the limited amount of data, of course, we cannot say if there is a disproportional contribution of the hippocampus to thinking about strangers or close others, but this remains an interesting question for future research.

It is likely that non-declarative forms of memory contribute to some aspects of empathy. Indeed, non-declarative forms of memory such as priming have been evoked to account for a range of social phenomena (Chartrand and Bargh, 1999; Milne and Grafman, 2001; Garrod and Pickering, 2009) and may contribute at some level to the complex set of processes that result in an empathic response. Understanding the interaction and time course of memory systems with other neural systems critical for social behavior will further our conceptualization of the network supporting the social brain.

While this study offers preliminary support for the notion that the hippocampus contributes to the neural network supporting empathy, there are certain caveats and limitations of the study that should be noted. The hippocampal patients did not report elevated levels of empathy following the induction protocol using two separate methods of empathy induction conducted on two separate occasions. However, these same induction procedures were successful in inducing empathy in healthy comparison participants (Beadle, 2011; Beadle et al., under review) and in individuals with frontal lobe damage in another study (Beadle, 2011). This result is also in contrast to our previous work inducing happiness and sadness in patients with hippocampal amnesia (Feinstein et al., 2010). Nevertheless, the amnesic patients did not report elevated empathy and thus we did not see an increase in prosocial behavior. While methodological differences in the induction protocols should be considered further, we are intrigued by the possibility that the hippocampus may not be required for the experience (and induction) of basic emotions but that it is required for social emotions such as empathy. Already there is some evidence that social emotions may recruit a different neural system than basic emotions (Moll et al., 2002). The hippocampus may be recruited for the processing of social information and emotions due to its role in relational thinking, i.e., thinking about others’ mental states and retrieving relevant autobiographical memories to understand others. Future work to explore this idea is warranted. The small sample size and the lack of familiar partner ratings of empathy from the healthy comparison participants are also limitations. Future replication and extension of this work is needed.

The goal of the current work was to argue for the inclusion of the hippocampus in the larger neural network supporting social behavior and empathy. Our preliminary results suggesting that hippocampal damage produces a deficit in empathy have implications for studying empathy in other populations where hippocampal pathology and declarative memory impairments are common (e.g., normal aging, Alzheimer’s Disease and other dementias, schizophrenia, and Traumatic Brain Injury). Indeed, because the hippocampus has not routinely been considered to be a significant part of the network critical for social behavior, such deficits are seldom attributed to the impairments in declarative memory that are a hallmark in so many patients that also have deficits in empathy.

## Conflict of Interest Statement

The authors declare that the research was conducted in the absence of any commercial or financial relationships that could be construed as a potential conflict of interest.

## Footnotes

^1^The healthy comparison participant matched to 2363 in Experiment 1 was no longer available at the time of Experiment 2, and thus a different healthy comparison participant that was matched to 2363 on demographic characteristics completed Experiment 2A.
